# Socioepidemiological macro-determinants associated with the cumulative incidence of bacterial meningitis: A focus on the African Meningitis Belt

**DOI:** 10.3389/fneur.2023.1088182

**Published:** 2023-02-14

**Authors:** Gabriel D. Pinilla-Monsalve, Natalia Llanos-Leyton, Maria Camila González, Edgar Fabian Manrique-Hernández, Juan José Rey-Serrano, Jairo Alonso Quiñones-Bautista

**Affiliations:** ^1^Departamento de Neurología, Fundación Valle del Lili, Cali, Colombia; ^2^Departamento de Ciencias Clínicas, Universidad Icesi, Cali, Colombia; ^3^Centre de Recherche, Institut Universitaire de Gériatrie de Montréal, Montréal, QC, Canada; ^4^Centro de Investigaciones Clínicas, Fundación Valle del Lili, Cali, Colombia; ^5^Departamento de Salud Pública, Universidad Industrial de Santander, Bucaramanga, Colombia; ^6^Facultad de Ciencias de la Salud, Universidad Autónoma de Bucaramanga, Bucaramanga, Colombia

**Keywords:** meningitis, African Meningitis Belt, poverty, temperature, socioepidemiological determinants

## Abstract

**Background:**

Bacterial meningitis (BM) is a public health challenge as it is associated with high lethality and neurological sequelae. Worldwide, most cases are registered in the African Meningitis Belt (AMB). The role of particular socioepidemiological features is essential for understanding disease dynamics and optimizing policy-making.

**Objective:**

To identify socioepidemiological macro-determinants that contribute to explaining the differences in BM incidence between AMB and the rest of Africa.

**Methods:**

Country-level ecologic study based on the cumulative incidence estimates of the Global Burden of Disease study and reports of the MenAfriNet Consortium. Data about relevant socioepidemiological features were extracted from international sources. Multivariate regression models were implemented to define variables associated with the classification of African countries within the AMB and the incidence of BM worldwide.

**Results:**

Cumulative incidences at the AMB sub-regions were 111.93 (west), 87.23 (central), 65.10 (east), and 42.47 (north) per 100,000 population. A pattern of common origin with continuous exposition and seasonality of cases was observed. Socio-epidemiological determinants contributing to differentiating the AMB from the rest of Africa were household occupancy (OR 3.17 CI 95% 1.09–9.22, *p* = 0.034) and malaria incidence (OR 1.01 CI 95% 1.00–1.02, *p* = 0.016). BM cumulative incidence worldwide was additionally associated with temperature and gross national income per capita.

**Conclusion:**

Socioeconomic and climate conditions are macro-determinants associated with BM cumulative incidence. Multilevel designs are required to confirm these findings.

## Introduction

Bacterial meningitis (BM) is still considered an event of interest in public health as it is related to high lethality rates and multiple neurological sequelae (1). This entity, among other infectious diseases, represents a relevant proportion of the burden of disease in low-income countries and is among the top causes of years lived with disability and premature death in both children and adults (2–4). This condition also has higher morbidity rates in vulnerable populations and at extreme ages (5). Likewise, BM represents a threat due to the potential severity of the clinical course, its tendency to generate outbreaks and epidemics in specific geographic regions (6), and the costs associated with its treatment [1.6 billion USD in 2006 (7), or up to 34% of the gross domestic product per capita (8)]. It is the sixth most lethal infection in the world and the third in newborns (9).

According to the GIDEON (Global Infectious Disease and Epidemiology Network), in 1997, there were 1,200,000 bacterial meningitis cases worldwide (41.66% in Africa) with an 11.35% lethality (10, 11). Over the last decades, high and upper-middle-income countries have relatively succeeded in reducing BM frequency by implementing standardized epidemiological surveillance systems and mass vaccination campaigns against the most frequent etiologic agents (3, 12). On the contrary, a region located in Sub-Saharan Africa, comprising more than twenty countries, is known as the African Meningitis Belt (AMB) because of the ongoing recurrence of BM outbreaks and epidemics. The Global Burden of Disease (GBD) estimates that almost 4 out of 5 cases in Africa are registered in this region. For 2016, this region accounted for >45% of cases worldwide (13).

Bacterial meningitis (mainly caused by *N. meningitidis, H. influenzae* type B, or *S. pneumoniae*), is a communicable disease associated with different climatological and social characteristics (14). In North America and Europe, BM cases peak during the winter (15–17), while in the AMB there is an evident seasonality as most patients demonstrate symptoms during the dry season (around the 6^th^ epidemiological week) (18, 19). Sub-Saharan Africa has high temperatures due to its relative distance to the Equator and geographical proximity to the desert, which also influences the air concentration of particulate matter (mineral dust) (20).

Studies addressing socioepidemiological features were mainly published between 1970 and 2000 and carried out in Europe (16, 21, 22), Oceania (23, 24), and Africa (25–27). In high-income countries, researchers like Haneberg et al. (28) identified distinct exposures including psychologically stressful events. During the meningococcus outbreak in Wales (2000), Fitzpatrick et al. evaluated the association of BM with psychological stressors including the death of a family member, moving home, traveling, and receiving bad news; after the multivariate adjustment, the latter remained associated with a meningococcus carrier state (22). In English preschoolers, BM incidence increased with recent exposure to dust, household overcrowding, and parental involvement in marital conflicts or legal issues (16). These results are consistent with associations found by Jones et al. regarding social deprivation, unemployment, and lack of house ownership (21).

Environmental exposures, for example, passive smoking (16, 23), alcohol abuse or dependence (29, 30), pacifier sharing (23), breastfeeding (24), and vitamin A supplementation (31) have also been proposed to be linked to BM.

Complementarily, Greenwood et al. analyzed a BM epidemic in Nigeria (1977) and suggested a possible association between incidence and socioeconomic status because few cases occurred in inhabitants with high-income (25). They also investigated variables such as social class, occupation, literacy, and nutritional state, among others, in the BM outbreak at The Gambia (1982) and found that the density of cattle was higher in regions that were not affected by the disease (26). This index has been considered a surrogate feature in the context of poverty (32). Subsequent publications have described the importance of social determinants of health on BM incidence but mainly from the individual scope. In 2001, Hodgson et al. applied a case-control design to analyze risk factors for meningococcal meningitis in survivors of the epidemic in the north of Ghana (1997); significant associations were found with exposure to smoke produced by firewood stoves and sharing the bedroom with a case (27).

Although different studies have already demonstrated that socioepidemiological conditions are associated with BM in individuals from the AMB, one could question why the cumulative incidence importantly diverges between two Sub-Saharan countries that share land borders. In other words, what are the unique conditions that the AMB populations face that make them more vulnerable to BM in contrast to the rest of Africa?

The main objective of this research was *(1)* to identify country-level statistics that contribute to explaining the differences in BM cumulative incidence between AMB and non-AMB African countries. For this purpose, we also *(a)* described the incidence of BM (attributed to *N. meningitidis, H. influenzae*, and *S. pneumoniae*) in the AMB compared to that in non-AMB African and non-African countries and *(b)* studied the epidemic curve per AMB subregion and the relationship with climate variables. As a secondary objective, we aimed *(2)* to determine if the same features were associated with the number of BM incident cases per country worldwide. We hypothesized that climate variables and country-level statistics related to poverty (i.e., unmet basic needs) were the main socioepidemiological macro-determinants of BM in both Africa and worldwide.

## Methods

This is a completely-ecological study with a multiple-group design that was conducted as a secondary analysis of aggregate, environmental, and global measures (BM incident cases in 2016 and country-level statistics) obtained mainly from the records of the GBD, the MenAfriNet Consortium, the World Bank and the World Health Organization (WHO).

According to Morgenstern, ecological studies “focus on the comparison of groups, rather than individuals” by analyzing aggregate (i.e., proportion of smokers), environmental (i.e., air-pollution levels), and global measures (i.e., population density) (33). Levels of analysis can be classified as completely-ecological (all variables are aggregate measures), partially-ecological (some joint distributions are known), and multilevel (both individual and aggregate measures of interest are available). There are three different designs relevant to ecological studies: multiple-group (“comparison of disease rates among regions during the same period”), time-trend (“comparison of disease rates over time in one geographically defined population”), and mixed study (a combination of the previous two) (33). A detailed description of ecological studies has been published elsewhere (34–36).

The main three advantages of ecological studies are the study of exposures of interest from the collective scope (social and environmental dynamics), the quick obtention of results at a low cost allowing preliminary decision-making in public health, and the generation of hypotheses that must be confirmed or refuted with further research (37).

### Unit of analysis

Countries reporting BM incident cases for 2016 were considered in the study (AMB *n* = 23, African countries outside the AMB *n* = 31, and countries from the other continents *n* = 130).

The first-level classification of countries within the AMB followed the definition of the MenAfriNet Consortium of areas at high epidemic risk (38). Burundi, Rwanda, and Tanzania were not included in the AMB for this study since they are considered countries with lower risk and not all bibliographic resources define them as part of the region. Second-level classification of AMB countries in subregions followed the United Nations geoscheme of Africa (39). For graphing the subregional epidemic curves and the bivariate and multivariate analyses, South Sudan was disregarded from the sample due to its recent independence in 2011 (40); there are dissimilarities in the different country-level statistics from external sources (some reporting only data from Sudan).

In the case of England, there was no consistency between the different sources as this country's territory is not equivalent to the United Kingdom (disaggregated information for Scotland, Wales, and Northern Ireland, was unavailable for several variables). Most of the variables could not be retrieved for Greenland.

### Country-level statistics

The region where the African country is located (inside or outside the AMB) was the outcome variable of the categorical regression model *(main objective)*. Categorization of African countries into these two groups has been previously proposed by Laxminarayan et al. (41), Mazamay et al. (1), and the GBD study (13).

The number of BM cases per 100,000 population reported by each country in 2016, was extracted from the GBD study and calculated after adding the incident cases of meningitis caused by *N. meningitidis, H. influenzae* type B, and *S. pneumoniae (secondary objective)*. The punctual location of each country was defined with latitude and longitude coordinates provided by Google Data Explorer. Besides, we calculated geohash (base-32) and transformed it into the decimal system (42).

Considering the literature about socioepidemiological features associated with BM, a set of 60 potential regressors was evaluated for each country including geo-environmental aspects, demographic characteristics, socioeconomic conditions, unmet basic needs, smoking and alcohol habits, nutritional variables, coverage of vitamin A supplementation, vaccination rates (immunization against *N. meningitidis* was not available for most countries), and additional causes of morbidity (for the operational definition of variables, and their source, see the Supplementary Table 1).

### Case definitions and primary data collection

The GBD estimates the incidence of infectious meningitis for each country *(specific objective a)*. Meningitis was defined as a “disease caused by inflammation of the meninges, the protective membrane surrounding the brain and spinal cord, and that is typically caused by an infection in the cerebrospinal fluid (CSF). Symptoms include headache, fever, stiff neck, and sometimes seizures” (13). Infectious meningitis is then classified into four groups: meningococcal, *H. influenzae* type B, pneumococcal, and others.

A systematic review of surveillance systems reports, scientific literature claims data-inpatient visits, and inpatient hospital data, published up to the end of 2013, was done. Cases were recorded with ICD-9 and ICD-10 codes: *N. meningitidis* (36-36.9 and A39-A39.9), *H. influenzae* (320 and G00.0), and *S. pneumoniae* (320.1 and G00.1). General incidence, and per infectious agent, were generated by Bayesian meta-regressions based on 1,348 non-fatal outcomes sources. To differentiate incident from prevalent cases, the lethality, rate of long-term complications, and sequelae fraction (epilepsy, vision impairment, hearing loss, motor and cognitive impairment, intellectual disability, and behavioral problems) were also computed (13).

The MenAfriNet Consortium and the WHO record suspected meningitis cases per epidemiological week (for the analysis of epidemic curves by subregion and when assessing the yearly trends of BM incidence in relationship with climate variables *(specific objective b)*, this is the level of certainty). Suspected cases are defined as: “any person with sudden onset of fever (>38.5°C rectal or 38°C axillary) and one of the following signs: neck stiffness, altered consciousness or other meningeal signs” and “any toddler with sudden onset of fever (>38.5°C rectal or 38°C axillary) and one of the following signs: neck stiffness, flaccid neck, bulging fontanel, seizure or other meningeal signs” (43).

Some of the cases underwent a lumbar puncture for confirmation in CSF. Basic cytochemical and microbiological analysis contribute to a probable level of certainty: “any suspected case with a macroscopic aspect of its CSF turbid, lousy or purulent; or with a microscopic test showing Gram-negative diplococcus, Gram-negative bacillus, Gram-positive diplococcus; or with leukocytes count more than 10 cells/mm^3^” (12, 43) and “any infant with CSF leukocyte count >100/mm^3^ or 10–100 cells/mm^3^ and either and elevated protein (100 mg/dL) or decreased glucose (< 40 mg/dL) level” (12, 43).

Finally, a smaller proportion reached the confirmed definition: “isolation or identification, in CSF or blood, of the causal pathogen (*N. meningitidis, H. influenzae* type B, *S. pneumoniae*, etc.) from the CSF of a suspected/probable case by culture, polymerase chain reaction, immunochromatographic dipstick or latex agglutination test” (12, 43).

Suspected cases are reported by providers at a health facility and informed to the district surveillance officer each week, who then compile and notify the data to the provincial and national instances. Notification must be done even in absence of cases and throughout the year. This information is merged by the national instance of each country and then sent to the WHO, and their partners, on a weekly or monthly basis (if no epidemic is registered). Laboratory tests' results must be also included (12, 43).

### Secondary data extraction and sources

Most of the country-level statistics did not need to be additionally coded, categorized, or transformed; as temperature, rainfall, and relative humidity are recorded by month, we calculated the median to obtain a yearly value. Data was retrieved by the first author from the official web pages of the different international organizations (primary sources of each country are not publicly available for validation). As stated before, BM yearly incident cases worldwide by country and weekly incident cases in the AMB by country were extracted from the GBD study and the MenAfriNet Consortium. Socioepidemiological country-level statistics were predominantly extracted from the World Bank and the WHO. For the description of every source studied, (see Supplementary information 1).

The GBD (11 variables) is an initiative that calculates specific morbidity, mortality, and disability rates associated with different diseases, injuries, and risk factors (44). The purpose is to “improve health systems and eliminate disparities” (45). Different sources (surveillance systems, scientific literature, inpatient claims, and hospital data) are examined through a systematic review and then modeled through Bayesian meta-regression techniques (13).

The MenAfriNet Consortium (2 variables) is an international partnership between the U.S. Center for Disease Control and Prevention, the WHO Regional Office for Africa, Davycas International, and some African Ministries of Health. Its purpose is to “evaluate the long-term effectiveness of existing vaccine programs and to support decision-making, implementation strategies, impact evaluations, and special studies for bacterial meningitis” in the AMB (38). Data is continuously recorded and transmitted from health facilities in each district to the international level as explained before.

The purpose of the World Bank DataBank (35 variables) is to collect data for “developing effective policies, monitor the implementation of poverty reduction strategies or progress toward global goals” in order to comply with the Marrakech Action Plan for Statistics (46). Before compilation, most data is provided by member countries that have developed official statistical systems at the national level. The World Bank specifies that the data quality depends on the accuracy of these systems. It also records information on climate variables such as temperature and rainfall through its Climate Change Group (47, 48).

The WHO (7 variables), in partnership with the United Nations International Children's Emergency Fund (UNICEF), informs national immunization coverage estimates that are calculated from “reported data and survey results”. Estimates of non-reporting countries are extrapolated from the last empirical data. The objective is to reduce “under-five mortality and monitor coverage of immunization services to guide disease control, elimination, and eradication” (49). The WHO-UNICEF instructs to “report routine immunization coverage using the number of doses administered” by service providers and only accounts for doses included in each national schedule. It also records data about vitamin A supplementation to prevent “blindness in children” and decrease “the risk of disease and death for severe infections” (50).

### Technical procedures

Choropleth maps for global BM incidence by the etiological agents were graphed using Microsoft Excel v.365. (Microsoft Corporation, Washington U.S.).

The MenAfriNet Consortium, in partnership with the WHO, publishes the surveillance data in the form of bulletins (.pdf documents) that include the epidemic curves per country. Hence, BM cases of AMB countries were extracted from the curve figures, using the automatized algorithm of WebPlotDigitizer, 3.11 (Automeris LLC, Texas U.S.). The yearly number of cases (sum of cases of every epidemiological week informed) obtained through this method was compared to the official consolidated number presented in the bulletin's table to guarantee its accuracy (100%, IQR 99.42–100%).

Epidemic curves by sub-region of the AMB were plotted from the obtained data and the wet season was marked between the 17^th^ and the 44^th^ epidemiological weeks (6).

### Statistical analysis

GBD cumulative incidences of BM (dependent variable) are expressed with a population base of 100,000 inhabitants. Country-level statistics are described with medians and interquartile ranges as the count of incident cases was not approximately normal (Shapiro-Wilk's *W* = 0.662, *p* = 0.000). Taking into account that the pattern of missing data in international reports is “not at random” (51), no statistical methods were implemented to handle this matter. Age structure indexes worldwide and latitude and longitude in Africa were correlated with BM incidences, per bacterial agent, with Spearman's coefficients (ρ). Differences between AMB countries and both non-AMB African countries and non-African countries were determined using the Kruskal-Wallis test with Dunn's *post hoc* test and Sidak's methodology for multiple comparisons adjustment.

Univariate logistic regressions were carried out to select variables that demonstrate *p*-value < 0.05 and McFadden's pseudo-*R*^2^ ≥0.10 (52), as measures of statistical significance and acceptable fit. Association with latitude, longitude, and/or geohash was also appraised. To assess for multicollinearity, factor analysis through the principal-component method (53, 54) was applied and variables with loadings (λ) >0.4 or < -0.4 (55) on the first factor were retained; subsequently, country-level statistics that had a variance inflation factor (VIF) < 5 (56) were set for the logistic multivariate modeling. Variable selection was done with the stepwise backward procedure until achieving significance for both the general model and each of its variables. Humidity was discarded from the regressions as it was missing for 32 African countries and is correlated with temperature and rainfall with higher VIF.

For the *secondary objective*, the same variables that were significant in the univariate logistic regression were considered as possible regressors of the BM incidence worldwide. Considering the potential over-dispersion of the dependent variable (various high-income countries exhibit very few BM incident cases), Poisson and negative binomial (with log link function) multivariate regressions were fitted following the same steps. The goodness of fit was evaluated with the Pearson and deviance residuals and specification with the link test.

Analyses were performed using Stata 14 (StataCorp, College Station, Texas U.S.) and R 4.1. (R Core Team, Vienna, Austria).

## Results

### Incidence of bacterial meningitis

Global BM incidence in 2016 was 13.47 (IQR 4.11–39.50) cases per 100,000 population. The AMB countries exhibited an incidence of 109.46 (IQR 57.50–166.94), which almost triples the frequency in the rest of Africa and is >17 times higher than in non-African countries. The country with the highest incidence was South Sudan (272.12), 1,700 times that of South Korea (0.16). BM incidences and vaccination rates are detailed in Table 1.

**Table 1:** Country-level statistics per country's region (outside Africa, inside Africa but not in the AMB, and in the AMB).

**Country-level statistics**	**Non-African (*n =* 130)**	**Non-AMB African (*n =* 31)**	**AMB (*n =* 23)**	**Total (*N =* 184)**
*Incidence of bacterial meningitis*	5.86 (2.44–15.92)	40.17 (23.41–55.64)	109.46 (57.5–166.94)	13.47 (4.11–39.5)
*Neisseria meningitidis* (100,000 population)	1.97 (1.1–5.08)	9.89 (7.42–12.37)	47.09 (20.96–101.35)	3.86 (1.35–10.53)
*Haemophilus influenzae* (100,000 population)	1.15 (0.31–4.19)	11.65 (5.52–18.38)	18.48 (12.91–31.24)	3.32 (0.46–9.03)
*Streptococcus pneumoniae* (100,000 population)	2.53 (0.73–6.26)	12.95 (8.07–20.61)	25.59 (12.15–36.82)	4.52 (1.58–12.25)
**Vaccination rates**				
*Haemophilus influenzae* type B (% 6 months)	95 (90–98)	92 (84–96)	90 (73–93)	94 (86–97)
*Streptococcus pneumoniae* (% 2 months)	96 (88–99)	97 (88–99)	95.5 (87.5–98)	96 (88–99)
*Streptococcus pneumoniae* (% 4 months)	94 (82.5–98)	92 (82–97)	91.5 (84–96)	93 (82–97)
*Streptococcus pneumoniae* (% 12 months)	91 (82–96)	90 (81–96)	89.5 (75.5–93)	91 (81–95)
**Possible socioepidemiological determinants**				
Annual median temperature (°C)	19.38 (9.73–26.08)	23.73 (22.28–25.3)	27.56 (25.67–29.11)	23.93 (11.68–26.39)
Gross birth rate (1,000 population)	15.38 (10.8–20.48)	31.79 (23.35–35.61)	36.84 (34.53–39.99)	19.35 (12.1–29.34)
Life expectancy (years)	75.22 (71.29–79.16)	63.64 (60.28–71.3)	59.68 (56.95–63.05)	73.04 (65.75–76.88)
Total fertility rate (birth per woman)	1.96 (1.62–2.51)	3.85 (2.77–4.72)	4.94 (4.6–5.59)	2.34 (1.74–3.84)
Female population 0–4 years (%)	7.55 (5.4–9.92)	14.27 (10.74–15.94)	16.02 (15.36–17.33)	9.37 (5.91–13.34)
Male population 0–4 years (%)	7.5 (5.77–10.06)	14.36 (10.96–16.23)	16.37 (15.56–18.04)	9.67 (6.25–13.78)
Population aged ≥65 (%)	8.14 (4.68–14.65)	3.13 (2.83–4.51)	2.94 (2.54–3.23)	5.79 (3.42–12.9)
Age dependency ratio (%)	50.85 (44.52–56.47)	68.79 (55.08–86.75)	85.62 (81.63–92.3)	53.3 (47.34–70.8)
Gross national income per capita (USD 2015)	8,440 (3,960–23,260)	2,675 (750–5,040)	805 (600–1,310)	5,190 (1,690–14,970)
Human development index adjusted for inequality	0.65 (0.54–0.77)	0.37 (0.32–0.45)	0.3 (0.27–0.34)	0.56 (0.39–0.71)
Household occupancy (members/dwelling)	3.5 (2.6–4.5)	4.45 (4.1–4.95)	5.4 (4.7–5.9)	3.9 (2.85–4.85)
Literacy rate (%)	96.3 (91.8–99.1)	79.4 (70.5–87.5)	55.5 (38.4–73.8)	92.6 (73.8–98.1)
Access to sanitation services (%)	93.2 (79.6–98.6)	47.7 (34.4–74.5)	21.3 (15.7–29)	85.6 (47.4–97.2)
Use of sanitation services (%)	94.45 (81.81–98.96)	43.79 (31.11–74.54)	21.95 (13.93–32.6)	87.17 (46.53–97.54)
Use of drinking water (%)	97.56 (92.31–99.7)	71.59 (56.71–86.46)	62.82 (45.84–69.61)	94.48 (74.97–98.95)
Access to clean methods for cooking (%)	97.07 (63.48–100)	30.87 (4.39–73.15)	5.93 (3.04–17.6)	81.88 (21.53–99.99)
Smoking in women (%)	12.4 (4.2–21.9)	3.8 (2.1–6)	1.05 (0.6–3.05)	7.75 (2.8–19.3)
Smoking in men (%)	34.6 (25.8–45.4)	31.4 (26.5–43.2)	21 (16.9–36.4)	32.4 (23.3–43.85)
Prevalence of anemia (% 6–59 months)	27.15 (21.4–33.1)	48.2 (36.7–60)	68.4 (58.6–73.7)	30.95 (25–47.95)
Incidence of malaria (1,000 population)	3.1 (0.45–15.8)	114.2 (14–215.1)	246 (97.6–348.8)	24.5 (2.7–173.7)
Incidence of hepatitis B (100,000 population)	290.81 (157.48–654.74)	1,797.95 (653.89–3,219.02)	3,679.54 (3,032.49–4,331.15)	481.2 (214.53–1,837.07)

Values are medians (IQR).

This table includes only the socioepidemiological macro-determinants that significantly differentiate (p < 0.050) countries within the AMB from the rest of Africa (results of the univariate logistic regression). Vaccination coverage for S. pneumoniae or H. influenzae was not associated with the region of the country. For the full list of country-level statistics, (see Supplementary Table 2).

In general, men have a higher BM incidence, which is more evident in the AMB countries (119.01 IQR 62.97–178.96 vs. 99.05 IQR 52.20–153.84 in women). Significant positive correlations were found between the percentages of women/men aged 0–4 years in each country and BM incidence regardless of the attributed agent (ρ > 0.76, *p* = 0.000). Conversely, there is a negative correlation with the percentage of inhabitants in each country that are aged ≥65 years (ρ < -0.75, *p* = 0.000).

**Figure 1:**
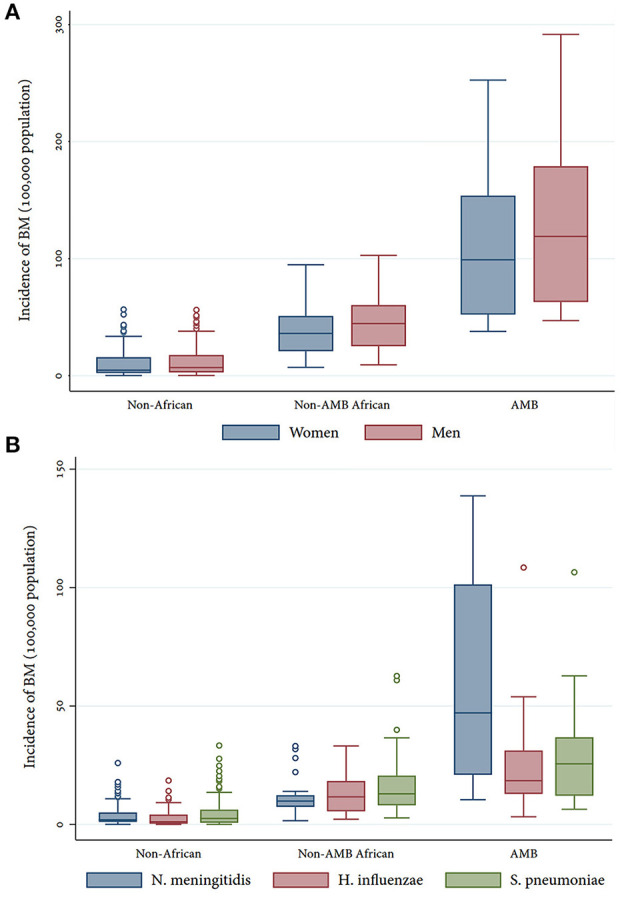
Incidence of BM by sex **(A)** and etiological agent **(B)** per region.

**Figure 2:**
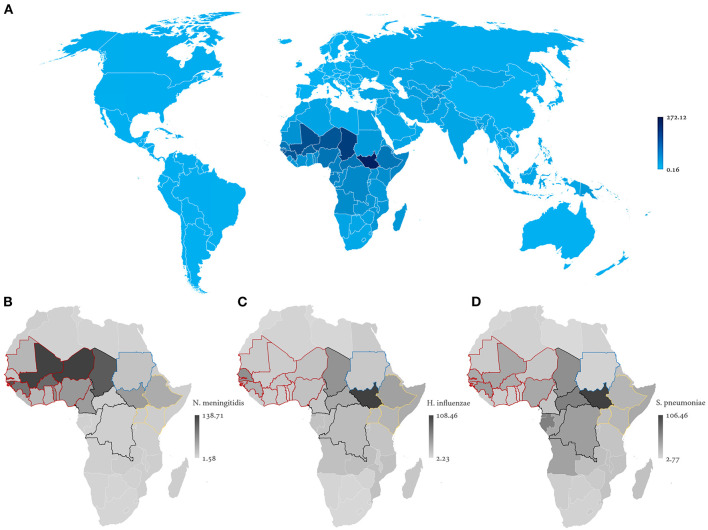
Geographical distribution of BM cumulative incidence per 100,000 population **(A)**, according to the etiological agent: *N. meningitidis* **(B)**, *H. influenzae* **(C)**, and *S. pneumoniae* **(D)**. Countries' land borders colored in red (west), black (central), blue (north), and yellow (east) delimit AMB sub-regions.

Incidences were comparable for the three main etiological agents except for the AMB where there is a clear predominance of *N. meningitidis* (Figure 1). The choropleth maps demonstrate a visual overlapping of the African countries with the highest incidence of BM attributed to *H. influenzae* and *S. pneumoniae* (center and east); on the contrary, cases of meningococcus are reported in regions closer to the Equator, with a tendency to be reported in western Africa (Figure 2). In this continent, latitude (ρ = 0.51, *p* = 0.001) and longitude (ρ = −0.51, *p* = 0.001) were only correlated with incident cases of meningococcal meningitis but not with those attributed to the other two bacteria.

BM incidence was higher in the AMB western subregion comprised of Benin, Burkina Faso, Ivory Coast, Ghana, Guinea, Mali, Mauritania, Niger, Nigeria, Senegal, Gambia, and Togo (111.93, IQR 68.44–163.67). The center (Cameroon, Central African Republic, Chad, and the Democratic Republic of the Congo, 87.23 IQR, 101.32–138.03) and east (Ethiopia, Kenia, and Uganda, 65.10, IQR 61.10–87.28) of the AMB demonstrated intermediate incidences, being lowest at the north (Sudan, 42.47).

**Figure 3:**
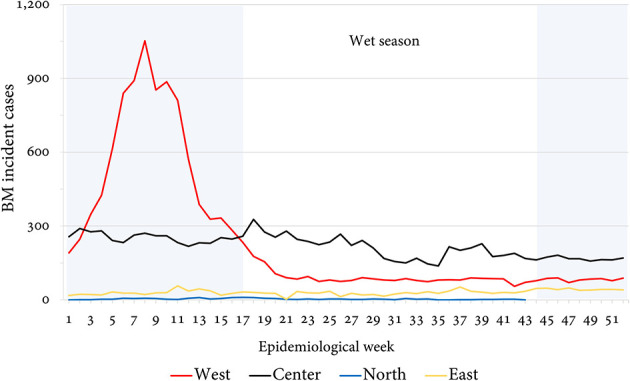
Report of incident cases by epidemiological week per AMB sub-region.

**Figure 4:**
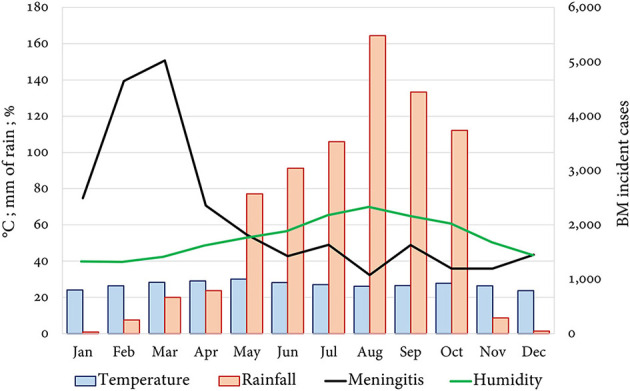
Report of incident cases and meteorological variables per month in the AMB.

According to the available epidemic curves of AMB countries, 13 (65%) completed the reports for every epidemiological week (52, 100%), while 7 showed partial reporting (epidemiological silence): Chad (22, 42.30%), Ethiopia (51, 98.07%), Gambia (42, 80.76%), Guinea (39, 75%), Mauritania (47, 90%), Sudan (43, 82.69%), and Uganda (17, 32.67%). In the epidemic curves by subregion, a continuous common source pattern is observed. The seasonality of cases (Figure 3) was higher in Togo (98% of cases in the dry season), Ghana (91%), and Nigeria (91%). From the climate scope, most cases over the year were recorded during months with higher temperatures and lower rainfall and humidity (Figure 4).

During 2016, lumbar puncture was performed in 42.71% of suspected BM cases, and of these, 71.90% of samples were negative for common pathogens. 82.73 and 7.08% of cases from the west and center subregions underwent a lumbar puncture, but no CSF specimens were collected for countries in the north or east regions.

### Description of socioepidemiological features

The full list of country-level statistics is described in Supplementary Table 2. The *post hoc* analysis demonstrated that countries within the AMB significantly differed (*p* < 0.050) from both non-AMB African countries and non-African countries in geo-environmental aspects (temperature, humidity, and air pollution), demographic characteristics (age dependency ratio), socioeconomic conditions (literacy and social conflicts generating displacement), consumption habits (tobacco smoking rate in men), unmet basic needs (access/use to clean cooking facilities and sanitation), and additional comorbidities (anemia, vitamin A deficiency, malaria, hepatitis B, and anxiety).

### Variables associated with a country's classification within the AMB

Results from univariate logistic regressions showed that 26 socioepidemiological macro-determinants were associated with the AMB category (*p* < 0.050) (Table 1). The highest and most significant odds ratios were found for variables related to the constructs of childhood and poverty: total fertility (OR 3.23 CI 95% 1.56–6.69, *p* = 0.002), household occupancy (OR 3.23 CI 95% 1.39–7.49, *p* = 0.006), population growth (OR 2.76 CI 95% 1.08–7.04, *p* = 0.034), temperature (OR 1.95 CI 95% 1.36–2.79, *p* = 0.000), and percentage of male preschoolers (OR 1.69 CI 95% 1.18–2.40, *p* = 0.004). Latitude (OR 1.06 CI 95% 1.02–1.11, *p* = 0.008) and longitude (OR 0.96 CI 95% 0.93–0.99, *p* = 0.017), but not decimal geohash (OR 1.00 CI 95% 0.99–1.00, *p* = 0.795), were also associated with the AMB classification.

The multivariate logistic model for differentiating countries located inside and outside the AMB (*n* = 41, pseudo-R^2^ = 0.31, *p* = 0.000) consists of household occupancy (OR 3.17 CI 95% 1.09–9.22, *p* = 0.034) and malaria cases per 1,000 population (OR 1.01 CI 95% 1.00–1.02, *p* = 0.016). Adjustment of the model seemed satisfactory according to Pearson's χ^2^ test result (*p* = 0.567), and an adequate specification was obtained with the included covariates (link error prediction *p* = 0.004, squared prediction *p* = 0.672). In this sense, an African country's probability of belonging to the AMB increases by 2.17 times for each additional member per dwelling and by 0.01 times for each extra case of malaria per 1,000 population.

### Variables associated with BM incidence worldwide

The final multivariate Poisson model (*n* = 77, pseudo-R^2^ = 0.62, *p* = 0.000) comprehends the incidence of malaria (*p* = 0.000), gross national income (GNI) per capita (*p* = 0.000), household occupancy (*p* = 0.000), and temperature (*p* = 0.000) (Table 2). Hence, BM cases per 100,000 population are reduced by < 0.01% when increasing the GNI per capita by 1 USD, from one country to another. On the opposite, the incidence of BM increases by 27.67, 4.58, and 0.34% when 1 extra member per dwelling, 1 additional Celsius degree, and 1 more case of malaria (per 1,000), are registered, respectively. However, when evaluating the goodness of fit of the model, it was observed that the Pearson and deviance residuals test results are significant (*p* = 0.000), suggesting that the BM incident cases count does not follow a Poisson distribution. The specification was also not satisfactory (link error prediction *p* = 0.000, squared prediction *p* = 0.000).

**Table 2:** Multivariate Poisson model for the incidence of BM in the world.

**Socioepidemiological determinants**	**IRR (CI 95%)**	**Error**	***p*-value**
Gross national income per capita (2015 USD)	0.999 (0.999–0.999)	< 0.001	< 0.001
Household occupancy (members/dwelling)	1.277 (1.240–1.314)	0.019	< 0.001
Annual median temperature (°C)^a^	1.035 (1.023–1.049)	0.006	< 0.001
Incidence of malaria (1,000 population)	1.003 (1.003–1.004)	0.000	< 0.001
Constant	3.629 (2.685-4.904)	0.557	< 0.001

^a^In the negative binomial regression, IRR 1.015 (CI 95% 0.946–1.091, p = 0.669).

A negative binomial regression (with a log link) was fitted with the same variables (pseudo-*R*^2^ = 0.109, *p* = 0.000), but the temperature did not remain significant (*p* = 0.534) after the multivariate adjustment. Considering the remaining three variables, the goodness of fit and specification were satisfactory (deviance residuals test *p* = 0.217, link error prediction *p* = 0.003, and squared prediction *p* = 0.067).

## Discussion

With this research, we analyzed socioepidemiological macro-determinants that possibly explain some of the differences between the AMB and non-AMB African countries. Our results support previous findings from studies at the individual and subnational levels, as similar variables were significant from the ecological scope. Population research conducted in specific AMB countries has shown 2.1 times higher incidence in areas with high population density, 34.8 times in those with very high levels of absolute poverty, and 41.6 times where the inhabitants exhibit low literacy rates (57). It has also been proposed that the use of closed kitchens with wood ovens, unhealthy housing, and low household income increase the likelihood of BM (27).

In middle-income countries from America, some other studies also recognize the relevance of socioepidemiological conditions on BM incidence. Molina et al. unveiled a possible association with air pollution sources in Cuba and reported higher incidences in municipalities with elevated population density and high percentages of infants and/or elders. These associations varied among cases attributed to *N. meningitidis, H. influenzae*, and *S. pneumoniae* (58). In Brazil, Fonseca et al. determined a relationship between BM incidence and poor living conditions (low income, housing in poor areas, literacy, access to drinking water, and household occupancy) (59). Except for the population density, the rest of the micro-determinants found in these studies were also significant at the country level whether in the bivariate *post hoc* or the multivariate analyses pursued here.

The population at the extremes of life has a greater susceptibility to developing BM due to the characteristics of their immune system. As synthesized by Simon et al., in newborns, the monocyte line is immature (60), which leads to impaired phagocytosis, inadequate response of natural-killer cells, poor cytokines and chemokines secretion, and limited concentrations of interferons. Moreover, the levels of complement factors are lower than in adults, and there is a high tolerance to self-antigens (61). In older adults, there is immune senescence characterized by absolute lymphopenia, decreased phagocytic and cytotoxic ability of neutrophils, and abnormal cytokine production, among others (62), etc.

In this sample, the main variables that make the AMB a distinct region and partially explained BM incidence worldwide were the temperature, gross national income per capita, household occupancy, and malaria incidence.

The annual median temperature in the AMB (27.56 °C, IQR 25.67–29.11) is not only higher than that in the other continents but also in the rest of Africa (~4 °C). Sub-Saharan African territories with high rates of BM demonstrate high temperatures and this seems related to their geographical location (intertropical convergence zone) (20). Likewise, it has suffered the impact of climate change due to the El Niño phenomenon (southern oscillation, ENSO), which favors the appearance of droughts (63); it is expected that the number of months in summer with temperatures above five standard deviations for the year 2100 would be 75% and up to 90% in west Africa (64).

Both temperature and rainfall are fundamental to understanding the dynamics of meningococcus since it has been demonstrated it can survive outside the human body, for up to 8 days, in glass, plastic, or cloth under favorable conditions of 30°C (65) and 22% relative humidity (66). The retrieved data show that temperature has a poor correlation with the volume of rainfall (ρ = 0.14, *p* = 0.052), while there is a clear relation with relative humidity (ρ = −0.29, *p* = 0.013). The association of this variable with the incidence of BM is also greater in comparison to that of rainfall (IRR 0.95 95% CI 0.93–0.96, *p* = 0.000 vs. 0.99, 95% CI 0.99–9.99, *p* = 0.000). This suggests that a fraction of the risk is attributed to low humidity beyond what is expected due to low volumes of rainfall during the dry season (Figure 4).

At the meteorological level, the beginning of an epidemic in the AMB coincides with a greater velocity of the dry winds (Harmattan) coming from the north and dragging mineral dust (a fraction of particulate matter 2.5) from the Sahara (6^th^±2 epidemiological weeks, *R*^2^ = 0.85) (18). This source of pollution is significantly concentrated in the AMB (40.04 μg/m^3^, IQR 32.68–50.05), where it is 1.5 times that of the rest of Africa. In Nigeria, the most populous AMB country, PM 2.5 was associated with 50,900 deaths (35,700–73,200) in 2015, while worldwide 675,000 (492,000–889,000) deaths attributed to lower respiratory tract infections were associated with air contaminants (67).

It is believed that detrimental changes in temperature, precipitation, and relative humidity favor colonization by meningococcus (68) and pneumococcus (69) after epithelial injury to the oral and nasopharyngeal mucosa, facilitating the translocation of the pathogen to the brain through the olfactory nerve. Mueller et al. synthesize the impact of the environment by proposing a model in which dusty weather increases bacterial invasiveness at the community level by up to 100 times (hyperendemicity) after the rainy season. Subsequently, an epidemic cofactor such as viral respiratory infection facilitates the carrier state and increases the incidence, generating localized epidemics during the dry season (70).

The relationship of BM incidence with the gross income per capita is an indicator of poverty and social deprivation as has been informed by multiple authors (21, 25, 57, 59). Intra-household and social overcrowding are also well-known determining factors that increase the risk of infections such as BM (27), tuberculosis, pneumonia, typhus, gastroenteritis, scabies, etc (71). In Auckland, Baker et al. found that two additional adolescents or adults in a 6-room house double the risk of children contracting meningococcal meningitis (23).

The incidence of malaria in west Africa increases during the dry season, similar to BM (72). Furthermore, it seems that higher temperatures increase the incidence of malaria since they facilitate the growth of the parasite and the survival of the infected mosquitoes (Anopheles sp.) (73). In the multivariate regressions, incident cases of BM were correlated with those of malaria, pointing to co-infection, as previously observed in Burkina Faso, where 11.8% of meningitis or bacteremia patients were infected as well by Plasmodium sp. (74). Both cases of cerebral malaria (*p* = 0.031) and BM (*p* = 0.014) have been associated with a deficiency of zinc levels compared to healthy controls from Ghana (75). We considered and collected the prevalence of zinc deficiency by country as an additional variable; in the univariate regression, it was associated with both the incidence of BM (IRR 1.05 CI 95% 1.04–1.05, *p* = 0.000) and malaria (IRR 1.04 CI 95% 1.03–1.04, *p* = 0.000). Unfortunately, this design is not adequate to define the impact of zinc deficiency as a common pathophysiological mechanism in these entities.

Given the low proportion of lumbar punctures, it could be possible that BM cases without a CSF microbiological study were actually caused by enteric pathogens whose route of transmission (fecal-oral) is related to the use/access to sanitation services (76) (an unmet basic need at the AMB). Particularly, group B streptococcus (5) is transmitted through this route and is considered the most common cause of BM in infants aged < 3 months (77). Even more, Berkman et al. carried out a study in Turkey, where 37% of specimens were positive for *Enterobacter* sp. or *Klebsiella* sp. while *S. pneumoniae* was found in only 6.5% (78), and in a series from Ghana, E. coli (3.4%), Salmonella sp. (3.4%), and Pseudomonas sp. (2.5%) were the most frequent bacteria after *S. pneumoniae* (77.7%) (79). Enterovirus can cause up to 80% of viral meningitis cases (54.6% of infectious meningitis) (80). From the populational perspective, improved sanitation and drinking water access are variables inversely associated with BM as a cause of death [see the GBD study (13)]. This has been also suggested in the past by Coulehan et al. who studied BM in Navajo Indians (81).

In this study, it was especially striking that immunization coverages against pneumococcus [effectiveness >90% (82)] were removed from the regression models since these are evident determinants of the individual risk for suffering BM. Unfortunately, we could not retrieve the coverage for the meningococcal vaccine of most high-income countries (83). Even though access to vaccination in Africa has increased by 38 times and the implementation of more than 20 mass immunization campaigns (268 million doses) has reduced the incidence of meningococcal serogroup A to a tenth, BM continues to be a critical public health problem (84). Due to the latent risk, the WHO and the Global Alliance for Vaccines and Immunization (GAVI) have determined that, as with cholera and yellow fever, it is necessary to increase the reserves of vaccines against meningococcus and ensure transportation logistics (85).

Regarding the study's limitations, it is not possible to make accurate inferences on the individual level due to the assumptions of the design (ecological fallacy). It was also complex to control for all potential confounders because important information (i.e., vaccination rates against *N. meningitidis*) were not available for every country. Also, the temporality of variables for every country might have been a source of bias (33, 36). Data was compiled from multiple sources, implying a variable information quality, and there was an important proportion of missing data as a minority of variables were available for every observation.

According to the MenAfriNet-WHO bulletins, lumbar puncture was performed for less than half of the AMB cases and it was not possible to reach a higher level of certainty about the etiological agent culprit of the clinical manifestations. This represents an information bias that depends solely on the available information. Similarly, the incident cases estimated by the GBD are calculated through meta-regression from different types of primary sources and it is also not possible to guarantee a microbiological confirmation. In the same sense, the calculation of BM incidence did not consider cases attributed to *Listeria monocytogenes* because this specific information is not recorded by the GBD; as this bacteria is responsible for an important number of cases in elders, future research will need to address this matter.

On the other hand, using multiple international databases allowed us to build a comprehensive panorama with diverse variables surrounding the BM phenomenon. By combining an evidenced-based rationale with a strict stepwise statistical analysis of multicollinearity, we attempted to reduce the effect of confounders [Simpson's paradox (86)]. The main strength of this study lies in addressing the BM public health challenge from a worldwide and continental ecological perspective by providing insights regarding a disease that has been mostly studied under traditional observational designs, which are susceptible to the individualistic fallacy. From this scope, the study confirmed most of the previous individual and national results and hopefully will inspire the development of novel hypotheses as some relevant determinants usually do not vary enough to be found as significant within populations, but they do when explored between populations (87). The results are also important for policy-making and reaffirm the relevance of social and structural determinants of health as its impact is clear on diseases such as BM that are “deadly, expensive and preventable” (38).

Future research should confirm or reject these findings by incorporating additional variables in multilevel designs that contribute to understanding better the link between epidemiological and pathophysiological mechanisms of BM.

## Conclusions

Consistent with the literature, these ecological results reinforce the relationship between a high cumulative incidence of BM in the AMB and socioepidemiological macro-determinants. Countries located within this region of Sub-Saharan Africa suffer from social inequity, poverty, unmet basic needs, low access to vaccines, and higher rates of other infections. From the environmental point of view, the AMB is characterized by high temperatures, low humidity, and increased levels of mineral dust contamination, which favor epidemic waves of the disease during the dry season, mainly in the western subregion. Modifiable socioepidemiological macro-determinants associated with BM cumulative incidence should be targeted by optimal public health policies.

## Data availability statement

The original contributions presented in the study are included in the article/Supplementary material, further inquiries can be directed to the corresponding author.

## Author contributions

GP-M: study conception and design, data collection, and analysis and interpretation of results. GP-M, NL-L, MG, EM-H, JR-S, and JQ-B: draft manuscript preparation. All authors reviewed the results and approved the final version of the manuscript.
