# Supplementary material for: Socioepidemiological macro-determinants associated with the cumulative incidence of bacterial meningitis: A focus on the African Meningitis Belt
Source: Front Neurol. 2023 Feb 14;14:1088182. doi: 10.3389/fneur.2023.1088182 (PMC9971970; doi:10.3389/fneur.2023.1088182)
Supplement: Supplementary file 1 [file Data_Sheet_1.pdf]

Supplementary material

Supplementary table 1. Variables definition and data sources.

Country-level statistics	Definition	Source and year
Incidence of bacterial meningitis	Cases of meningitis (N. meningitidis, H. influenzae, S. pneumoniae) per 100,000 population	Global Burden of Disease, 2016
Incidence of meningococcal meningitis	Cases of meningitis due to N. meningitidis per 100,000 population	Global Burden of Disease, 2016
Incidence of H. influenzae meningitis	Cases of meningitis due to H. influenzae B per 100,000 population	Global Burden of Disease, 2016
Incidence of pneumococcal meningitis	Cases of meningitis due to S. pneumoniae per 100,000 population	Global Burden of Disease, 2016
Incidence in men	Cases of meningitis in men per 100,000 men	Global Burden of Disease, 2016
Incidence in women	Cases of meningitis in women per 100,000 women	Global Burden of Disease, 2016
Geographic region and sub-region	Classification outside Africa, inside Africa but not in the AMB, and in the AMB	MenAfriNet Consortium, 2022
Geo-environmental aspects		
Latitude	Coordinate specifying the north-south distance of the Equator	Google Public Data Explorer, 2012
Longitude	Coordinate specifying the east-west distance of the Greenwich Meridian	Google Public Data Explorer, 2012
Temperature	Annual median temperature in centigrade degrees	World Climate Change Group, 2016
Rainfall	Annual median rainfall in millimeters of rain	World Climate Change Group, 2016
Relative humidity	Annual median relative humidity in percentage	World Meteorological Organization, 1961–1990
Land dedicated to agriculture	Percentage of the landscape dedicated to agriculture	World Bank, 2016
Cattle	Number of heads of cattle per 1,000 population	Food and Agriculture Organization, 2016
Pollution	Average annual exposure to PM 2.5 per cubic meter of air	World Bank, 2016
Demographic characteristics		
Total population	Number of inhabitants in the country	World Bank, 2016
Population density	Number of inhabitants per square kilometer	World Bank, 2016
Population growth	Percentage of annual population growth	World Bank, 2016
Rural population growth	Percentage of annual rural population growth	World Bank, 2016
Rural population	Percentage of rural population	World Bank, 2016
Female population	Percentage of women	World Bank, 2016
Sex ratio at birth	The ratio of male neonates	World Bank, 2016
Gross birth rate	Number of live births per 1,000 population	World Bank, 2016
Life expectancy	Number of years a person can expect to live	World Bank, 2016
Total fertility rate	Number of births per woman	World Bank, 2016
Adolescent fertility rate	Number of births per 1,000 women aged 15-19 years	World Bank, 2016
Pre-school population	Percentage of the population aged 0-4 years	World Bank, 2016
Elderly population	Percentage of the population aged ≥65 years	World Bank, 2016
Age dependency ratio	Proportion between dependent (<15 & >65 years) and active populations (15-64 years)	World Bank, 2016
Socioeconomic conditions		
Gross national income per capita	Gross national income in USD (after applying the Atlas conversion factor) per inhabitant	World Bank, 2015
Population under the line of poverty	Percentage of the population with income below that considered sufficient for their country	U.S. CIA, 2010 (IQR 2006-2012)
Adjusted human development index	Human development index adjusted by the Atkinson family of inequality measures	United Nations Development Program, 2015
Household occupancy	Number of members per dwelling	United Nations, 2011 (IQR 2010-2012)
Overcrowding	Percentage of population in overcrowding (>2 members per room)	Infographic by the Universities of Sheffield and Michigan based on World Bank data, 2002
Literacy rate	Proportion of people aged ≥15 years who can read and write	United Nations, 2015
Unemployment rate	Proportion of productive population that was unemployed	World Bank, 2016
Female workforce	Proportion of women in the labor force	World Bank, 2016
Displacement	Newly displaced persons due to social conflicts-natural disasters per 100,000 population	Internal Displacement Monitoring Center, 2016

Country-level statistics	Definition	Source and year
Basic needs		
Access to sanitation services	Percentage of the population with access to sanitary services (sewer network or septic tank)	World Bank, 2016
Use of sanitation services	Percentage of the population that uses sanitary services (sewer network or septic tank)	World Bank, 2015
Open defecation	Proportion of the population that practices open defecation	World Bank, 2016
Access and use of drinking water	Percentage of the population with access and that drinks clean water	World Bank, 2016
Access to clean methods for cooking	Proportion of the population with access to clean technologies for cooking	World Bank, 2015
Consumption habits		
Smoking in women	Proportion of women that smokes tobacco	World Bank, 2016
Smoking in men	Proportion of men that smokes tobacco	World Bank, 2016
Alcohol drinking	Average annual consumption of liters of pure alcohol per capita in those aged ≥15 years	World Bank, 2016
Nutritional variables		
Low weight at birth	Percentage of live neonates with low weight at birth	World Bank, 2016
Exclusive breastfeeding	Percentage of children exclusively breastfed during the first 6 months of life	Global Nutrition Monitoring Framework, 2012
Prevalence of undernourishment	Proportion of the population in a state of undernourishment	World Bank, 2016
Coverage of supplements and vaccination		
Vitamin A supplementation (≤59 months)	Proportion of children aged ≤59 months who had received vitamin A supplementation	World Health Organization, 2016
Immunization against tuberculosis (birth)	Proportion of newborns immunized against tuberculosis at birth	World Health Organization, 2016
Imm. diphtheria-tetanus-pertussis (2 months)	Proportion of 2-month children immunized against *C. diphtheriae*, *B. pertussis*, and *C. tetani*	World Health Organization, 2016
Immunization against hepatitis B (birth)	Proportion of newborns immunized against Hepatitis B Virus at birth	World Health Organization, 2016
Imm. against poliomyelitis (2 months)	Proportion of 2-month children immunized against Poliovirus	World Health Organization, 2016
Imm. H. influenzae type B (6 months)	Proportion of 6-month children immunized against *Haemophilus influenzae* type B	World Health Organization, 2016
Imm. S. pneumoniae (2, 4, and 12 months)	Proportion 2-, 4-, and 12-month children immunized against *Streptococcus pneumoniae*	World Health Organization, 2016
Concomitant morbidity		
Prevalence of anemia	Percentage of children aged 6-59 months with iron deficiency anemia, defined as Hb <110 g/L	World Bank, 2016
Incidence of hypovitaminosis A	Cases of vitamin A deficiency per 100,000 population	Global Burden of Disease, 2016
Incidence of malaria	Cases of malaria per 1,000 population	World Bank, 2016
Incidence of tuberculosis	Cases of tuberculosis per 100,000 population	World Bank, 2016
Incidence of Hepatitis B	Cases of acute hepatitis B per 100,000 population	Global Burden of Disease, 2016
Incidence of HIV/AIDS	Cases of HIV/AIDS per 100,000 population	Global Burden of Disease, 2016
Incidence of depression	Cases of depressive disorders per 100,000 population	Global Burden of Disease, 2016
Incidence of anxiety	Cases of anxiety disorders per 100,000 population	Global Burden of Disease, 2016

U.S. CIA: Central Intelligence Agency of the United States of America; Imm: Immunization.

Supplementary table 2. Full list of country-level statistics per country's region.

Country-level statistics	Non-African (n=130)	African non-AMB (n=31)	AMB (n=23)	Total (N=184)
Incidence of bacterial meningitis	5.86 (2.44-15.92)	40.17 (23.41-55.64)	109.46 (57.50-166.94)	13.47 (4.11-39.50)
Neisseria meningitidis (100,000 population)	1.97 (1.10-5.08)	9.89 (7.42-12.37)	47.09 (20.96-101.35)	3.86 (1.35-10.53)
Haemophilus influenzae (100,000 population)	1.15 (0.31-4.19)	11.65 (5.52-18.38)	18.48 (12.91-31.24)	3.32 (0.46-9.03)
Streptococcus pneumoniae (100,000 population)	2.53 (0.73-6.26)	12.95 (8.07-20.61)	25.59 (12.15-36.82)	4.52 (1.58-12.25)
Incidence in women (100,000 population)	4.69 (2.29-15.8)	36.15 (20.95-51.04)	99.05 (52.20-153.84)	12.64 (3.38-36.63)
Incidence in men (100,000 population)	7.00 (2.79-17.69)	44.53 (25.22-60.34)	119.01 (62.97-178.96)	14.64 (4.96-42.36)
Geo-environmental aspects				
Annual median temperature (°C)	19.38 (9.73-26.08)	23.73 (22.28-25.30)	27.56 (25.67-29.11)	23.93 (11.68-26.39)
Annual median rainfall (mm^3)	69.57 (42.00-117.25)	33.33 (17.80-121.75)	55.93 (7.25-94.91)	65.94 (30.72-112.87)
Annual median relative humidity (%)	72.71 (65.95-78.00)	72.63 (64.93-79.00)	54.38 (38.49-63.08)	71.41 (61.50-77.75)
Land dedicated to agriculture (%)	37.22 (20.45-52.46)	47.48 (28.03-71.05)	45.15 (33.77-64.78)	40.39 (21.84-57.70)
Heads of cattle (1,000 population)	40.26 (13.46-62.76)	19.81 (6.05-51.80)	35.03 (17.17-62.41)	34.02 (13.32-58.28)
Average annual exposure to PM 2.5 ($\mu g/m^3$)	19.99 (13.72-29.06)	24.70 (19.54-44.79)	40.04 (32.68-50.05)	22.21 (15.27-37.78)
Demographic characteristics				
Population density (inhabitants/Km^2)	81.98 (24.84-154.74)	47.90 (22.82-133.89)	64.57 (16.32-102.40)	74.70 (24.20-142.69)
Population growth (%)	1.01 (0.33-1.60)	2.28 (1.66-2.88)	2.71 (2.54-3.00)	1.30 (0.51-2.35)
Rural population growth (%)	0.03 (-0.73-0.90)	1.52 (0.57-2.10)	1.75 (1.38-2.34)	0.48 (-0.41-1.60)
Rural population (%)	33.96 (20.75-54.46)	56.87 (34.91-67.79)	60.00 (50.67-74.38)	42.58 (24.70-61.24)
Female population (%)	50.39 (49.65-51.05)	50.47 (49.80-50.90)	50.11 (49.92-50.27)	50.26 (49.78-50.88)
Sex ratio at birth	1.05 (1.05-1.06)	1.03 (1.03-1.05)	1.04 (1.03-1.05)	1.05 (1.04-1.06)
Gross birth rate (1,000 population)	15.38 (10.80-20.48)	31.79 (23.35-35.61)	36.84 (34.53-39.99)	19.35 (12.10-29.34)
Life expectancy (years)	75.22 (71.29-79.16)	63.64 (60.28-71.30)	59.68 (56.95-63.05)	73.04 (65.75-76.88)
Total fertility rate (birth per woman)	1.96 (1.62-2.51)	3.85 (2.77-4.72)	4.94 (4.60-5.59)	2.34 (1.74-3.84)
Adolescent fertility rate (1,000 women 15-19 years)	22.73 (9.68-48.68)	76.21 (30.97-108.94)	92.06 (76.88-122.28)	38.88 (13.84-71.29)
Female population 0-4 years (%)	7.50 (5.77-10.06)	14.36 (10.96-16.23)	16.37 (15.56-18.04)	9.67 (6.25-13.78)
Male population 0-4 years (%)	7.55 (5.40-9.92)	14.27 (10.74-15.94)	16.02 (15.36-17.33)	9.37 (5.91-13.34)
Population aged ≥65 (%)	8.14 (4.68-14.65)	3.13 (2.83-4.51)	2.94 (2.54-3.23)	5.79 (3.42-12.90)
Age dependency ratio (%)	50.85 (44.52-56.47)	68.79 (55.08-86.75)	85.62 (81.63-92.30)	53.30 (47.34-70.80)
Socioeconomic conditions				
Gross national income per capita (USD 2015)	8440 (3960-23260)	2675 (750-5040)	805 (600-1310)	5190 (1690-14970)
Population under the line of poverty (%)	19.50 (11.50-29.90)	53.00 (23.00-64.00)	43.40 (39.00-50.00)	24.90 (14.30-41.00)
Human development index adjusted for inequality	0.65 (0.54-0.77)	0.37 (0.32-0.45)	0.30 (0.27-0.34)	0.56 (0.39-0.71)
Household occupancy (members/dwelling)	3.50 (2.60-4.50)	4.45 (4.10-4.95)	5.40 (4.70-5.90)	3.90 (2.85-4.85)
Overcrowding (%)	26 (21-37)	35 (31-38)	38 (35-38)	29 (21-37)
Literacy rate (%)	96.30 (91.80-99.10)	79.40 (70.50-87.50)	55.50 (38.40-73.80)	92.60 (73.80-98.10)
Unemployment rate (%)	6.50 (4.40-10.80)	10.65 (6.00-18.20)	6.15 (4.30-9.80)	6.50 (4.40-11.20)
Female workforce (%)	42.95 (38.55-46.53)	45.72 (39.32-48.92)	46.33 (43.47-48.96)	43.81 (39.25-47.20)
Total displacement (100,000 population)	2.11 (0.00-106.99)	0.00 (0.00-66.30)	79.57 (0.00-354.03)	3.67 (0.00-107.56)
Displacement due to social conflicts (100,000 pop.)	0.00 (0.00-0.00)	0.00 (0.00-0.00)	0.00 (0.00-269.46)	0.00 (0.00-0.00)
Displacement due to natural disasters (100,000 pop.)	0.32 (0.00-59.24)	0.00 (0.00-52.62)	8.83 (0.00-95.83)	0.45 (0.00-60.77)

Country-level statistics	Non-African (n=130)	African non-AMB (n=31)	AMB (n=23)	Total (N=184)
Basic needs				
Access to sanitation services (%)	93.20 (79.60-98.60)	47.70 (34.40-74.50)	21.3 (15.70-29.00)	85.60 (47.40-97.20)
Use of sanitation services (%)	94.45 (81.81-98.96)	43.79 (31.11-74.54)	21.95 (13.93-32.60)	87.17 (46.53-97.54)
Open defecation (%)	1.62 (0.26-6.97)	11.26 (3.03-30.04)	23.92 (12.08-50.68)	5.23 (0.63-19.98)
Access to drinking water (%)	98.15 (93.25-100.00)	83.60 (74.10-93.20)	72.05 (58.20-79.00)	95.10 (81.90-99.60)
Use of drinking water (%)	97.56 (92.31-99.70)	71.59 (56.71-86.46)	62.82 (45.84-69.61)	94.48 (74.97-98.95)
Access to clean methods for cooking (%)	97.07 (63.48-100.00)	30.87 (4.39-73.15)	5.93 (3.04-17.60)	81.88 (21.53-99.99)
Consumption habits				
Smoking in women (%)	12.40 (4.20-21.90)	3.80 (2.10-6.00)	1.05 (0.60-3.05)	7.75 (2.80-19.30)
Smoking in men (%)	34.60 (25.80-45.40)	31.40 (26.50-43.20)	21.00 (16.90-36.40)	32.40 (23.30-43.85)
Alcohol drinking (liters/pop.-year)	6.60 (3.10-10.20)	5.80 (1.90-8.10)	3.60 (1.40-5.40)	6.10 (2.40-9.10)
Nutritional variables				
Low weight at birth (%)	8.30 (6.10-11.00)	12.90 (9.90-14.00)	14.00 (11.00-18.00)	9.60 (6.90-12.00)
Exclusive breastfeeding (%)	31.25 (16.90-42.70)	40.35 (16.20-60.40)	43.25 (26.90-52.50)	32.85 (18.00-49.00)
Prevalence of undernourishment (%)	4.40 (2.50-10.00)	19.60 (7.00-30.90)	13.45 (9.10-26.95)	6.35 (2.50-15.30)
Coverage of supplements and vaccination				
Vitamin A supplementation (% ≤59 months)	79 (62-96)	63 (47-75)	51 (29-95)	72 (50-92)
Imm. tuberculosis (% at birth)	96 (89-99)	94 (90-99)	92 (79-97)	96 (88-99)
Imm. diphtheria-tetanus-pertussis (% 2 months)	97 (93-99)	97 (89-99)	94 (84-98)	97 (92-99)
Imm. hepatitis B (% at birth)	91 (68-98)	90 (85-99)	58 (36-64)	91 (67-98)
Imm. *Haemophilus influenzae* type B (% 6 months)	95 (90-98)	92 (84-96)	90 (73-93)	94 (86-97)
Imm. poliomyelitis (% 2 months)	95 (81-99)	79 (46-96)	68 (59-77)	93 (70-98)
Imm. *Streptococcus pneumoniae* (% 2 months)	96 (88-99)	97 (88-99)	96 (88-98)	96 (88-99)
Imm. *Streptococcus pneumoniae* (% 4 months)	94 (83-98)	92 (82-97)	92 (84-96)	93 (82-97)
Imm. *Streptococcus pneumoniae* (% 12 months)	91 (82-96)	90 (81-96)	90 (76-93)	91 (81-95)
Concomitant morbidity				
Prevalence of anemia (% 6-59 months)	27.15 (21.40-33.10)	48.20 (36.70-60.00)	68.40 (58.60-73.70)	30.95 (25.00-47.95)
Incidence of hypovitaminosis A (100,000 population)	1009.83 (356.93-3363.12)	7326.56 (2052.79-23718.46)	20258.53 (10249.18-36164.26)	1831.91 (485.42-7464.83)
Incidence of malaria (1,000 population)	3.10 (0.45-15.80)	114.20 (14.00-215.10)	246.00 (97.60-348.80)	24.50 (2.70-173.70)
Incidence of tuberculosis (100,000 population)	26 (9-79)	242 (74-379)	159 (88-212)	52 (15-172)
Incidence of hepatitis B (100,000 population)	290.81 (157.48-654.74)	1797.95 (653.89-3219.02)	3679.54 (3032.49-4331.15)	481.20 (214.53-1837.07)
Incidence of HIV/AIDS (100,000 population)	6.63 (2.37-12.10)	59.55 (12.87-384.25)	56.7 (36.15-115.69)	10.51 (3.49-32.72)
Incidence of anxiety (100,000 population)	573.83 (519.26-654.69)	541.46 (519.60-587.95)	504.38 (501.00-524.32)	544.77 (507.89-647.83)
Incidence of depression (100,000 population)	3562.92 (3026.24-4150.41)	3594.46 (3186.16-4261.26)	3279.59 (3056.07-3509.96)	3476.13 (3059.79-4135.18)

Values are medians (IQR). Imm: Immunization; pop: population.

Supplementary information 1. Description of data sources (number of variables).

- The **GBD** (11 variables) is an initiative that calculates specific morbidity, mortality, and disability rates associated with different diseases, injuries, and risk factors[44]. The purpose is to "improve health systems and eliminate disparities"[45]. Different sources (surveillance systems, scientific literature, inpatient claims, and hospital data) are examined through a systematic review and then modeled through Bayesian meta-regression techniques[13].

- The **MenAfriNet Consortium** (2 variables) is an international partnership between the US Center for Disease Control and Prevention, the WHO Regional Office for Africa, Davycas International, and African Ministries of Health. Its purpose is to "evaluate the long-term effectiveness of existing vaccine programs and to support decision-making, implementation strategies, impact evaluations, and special studies for bacterial meningitis" in the AMB[38]. Data is continuously recorded and transmitted from health facilities in each district to the international level as explained before.

- The **World Bank** DataBank was the primary source of socioepidemiological country-level statistics (35 variables). The purpose of collecting this data is to "develop effective policies, monitor the implementation of poverty reduction strategies or progress towards global goals" in order to comply with the Marrakech Action Plan for Statistics[46]. Before compilation, most data is provided by member countries that have developed official statistical systems at the national level. The World Bank specifies that the data quality depends on the accuracy of these systems[46]. It also records information regarding climate variables such as temperature and rainfall through its Climate Change Group[47,48].

- The **WHO** (7 variables), in partnership with the United Nations International Children's Emergency Fund (UNICEF), informs national immunization coverage estimates that are calculated from "reported data and survey results". Estimates of non-reporting countries are extrapolated from the last empirical data. The objective is to reduce "under-five mortality and monitor coverage of immunization services to guide disease control, elimination, and eradication"[49]. The WHO-UNICEF instructs to "report routine immunization coverage using the number of doses administered" by service providers and only accounts for doses that are included in each national schedule. It also records data about vitamin A supplementation to prevent “blindness in children" and decrease "the risk of disease and death for severe infections"[50].

- The **Internal Displacement Monitoring Centre** (3 variables) collects data to inform operational plans of humanitarian responses and performs analysis and validation of internal displacement to understand its causes and triggers[88]. Data is gathered from national governments, local authorities, the United Nations and other international organizations, civil society organizations, research institutions, national Red Cross and Red Crescent societies, and the private sector. In addition, information is also retrieved from media monitoring and uses satellite imagery, natural language processing, and machine-learning techniques[88] .

- The **Google Public Data Explorer** (2 variables) is a collaboration with the Pardee Center[89] to access, explore and visualize large datasets and public databases[90]. Latitude and

longitude[91] are extracted from Google's geographical information systems using an eXtensible Markup Language (XML) function (geo)[92].

- The **Central Intelligence Agency** (1 variable) publishes the World Factbook to "provide basic intelligence"[93] for 266 world entities and was "designed for government officials and policymakers"[94]. It is considered an authoritative resource because the Information is provided by the U.S. Departments of Defense, Commerce, Labor, Energy, State, Interior, and Transportation. It also gathers information from the Central Intelligence Agency itself, the National Science Foundation, the United Nations Population Division, the International Telecommunication Union, the International Institute for Strategic Studies, Oil Gas Journal, among others[93]. The Factbook is considered a "comprehensive and valuable tool in country studies research"[94] and has been used as a source of reliable data by multiple investigations in health sciences[95–98].

- The **Global Nutrition Monitoring Framework** (1 variable) from the WHO provides information regarding breastfeeding as part of its goal to "improve maternal, infant and young child nutrition" and to "monitor the progress of primary and intermediate outcome, programs, and policies"[99]. Data comes from the Data Bank on Infant and Young Child Feeding which compiles information from "national and regional surveys, and studies about (...) the prevalence and duration of breastfeeding". Data is checked and validated afterwards[100].

- The **United Nations Educational, Scientific and Cultural Organization** (1 variable) projects literacy rates per country to comply with Millennium Development Goals and the Education for All (EFA) objective to "reduce illiteracy rates by 50%" when comparing 2000 and 2015[101]. Information is gathered by each country "through population censuses or household surveys in which the respondent or head of the household declares whether they can read and write (...); some surveys require (the individual) to take a quick test"[102].

- The **United Nations Department of Economic and Social Affairs** (Population Division) (1 variable) generates and analyses "economic, social and environmental data" of states members. Its purpose with the data is to "strengthen the capacity (...) to monitor population trends and address current and emergent population issues"[103]. Among these, they inform the household size and composition. Data is pulled from five sources: Demographic and Health Surveys (74 countries), Multiple Indicator Cluster Surveys (97 countries), the Minnesota Population Center (93 countries), the Demographic Yearbook (126 countries), and the Labor Force Surveys (24 countries). Details about the household are usually reported by the household head[103,104].

- The **United Nations Development Program** (1 variable) is an agency that works to "eradicate poverty and reduce inequality" by helping "countries to develop policies, leadership, skills, abilities, capabilities and to build resilience to achieve the Sustainable Development Goals"[105]. It is focused as well on assessing human development as a way to "expand the richness of human life rather than simply the richness of the economy". The index "was created to emphasize that people and their capabilities should be the ultimate criteria for assessing the development of a country and not the growth alone" and also, can be "used to question national policy choices"[106]. It is calculated with 4 indicators and 3 health,

education, and standards of living dimension indexes published by international partners (i.e., World Bank, WHO, United Nations, etc.[107]).

- The **United Nations Food and Agriculture Organization** (1 variable) is the main authority for the worldwide collection, verification, and communication of data related to food agriculture[108]. It performs face-to-face interviews by trained enumerators with holders from agricultural production units located worldwide to obtain data[109] (including livestock units) which is key in the development of policies and to inform decision-makers to approach issues related to hunger and malnutrition, rural poverty, use of natural resources, climate change, among others[110].

- The **World Meteorological Organization** (1 variable) collects data for "better understanding the climatology of severe weather and extreme events (...) to save lives and property and improving understanding and monitoring of the climate system and environment"[111]. The main purpose of the data is to address "major challenges such as food security, water resources management, reduction of pollution, decision-making related to the health sector", etc. Information is collected from several weather stations located worldwide[111,112].

Additional references:

88. Internal Displacement Monitoring Center. Displacement data sources. *Monitoring Tools*, https://www.internal-displacement. org/monitoring-tools (2022).
89. Pardee School of Global Studies. About the center. *Study of the Longer-Range Future*, https://www.bu.edu/pardee/about/ (2022).
90. Pardee Center for International Future. Google Public Data Explorer. *Research Projects*, https://korbel.du.edu/research/project/googles-public-data-explorer (2022).
91. Google Developers. Guides: countries.csv. *Dataset Publishing Language*, https://developers.google.com/public-data/docs/canonical/countries_csv (2012).
92. Google Developers. XML function: geo. *Dataset Publishing Language*, https://developers.google.com/public-data/docs/canonical/geo_xml (2012).
93. Central Intelligence Agency. *The World Factbook*. Washington D.C.: Office of Public Affairs, https://www.cia.gov/the-world-factbook/ (2021).
94. Geck C. The World Factbook. *Charlest Advis* 2017; 19: 58–60.
95. Wolff RF, Reinders S, Barth M, et al. Distribution of country of origin in studies used in Cochrane Reviews. *PloS One* 2011; 6: e18798.
96. Grasgruber P, Cacek J, Kalina T, et al. The role of nutrition and genetics as key determinants of the positive height trend. *Econ Hum Biol* 2014; 15: 81–100.
97. Grasgruber P, Sebera M, Hrazdíra E, et al. Major correlates of male height: A study of 105 countries. *Econ Hum Biol* 2016; 21: 172–195.
98. Bowles BC, Gibson M, Jansen L. The childbirth educator’s role in teaching post-birth warning signs. *J Perinat Educ* 2020; 29: 90–94.
99. World Health Organization. Global Nutrition Monitoring Framework. *Nutrition Landscape Information System*, https://www.who.int/data/nutrition/nlis/gnmf (2022).
100. World Health Organization. Infants exclusively breastfed for the first six months of life. *Global Health Observatory*, https://www.who.int/data/gho/indicator-metadata-registry/imr-details/3144 (2022).
101. United Nations Educational, Scientific and Cultural Organization. *Adult and Youth Literacy*. Paris: Institute for Statistics, http://uis.unesco.org/sites/default/files/documents/fs32-adult-and-youth-literacy-2015-en_0.pdf (2015).
102. United Nations Educational, Scientific and Cultural Organization. Education and literacy. *Statistical Topics*, http://uis.unesco.org/en/topic/literacy (2022).

103. United Nations. *Database on Household Size and Composition*. Washington D.C.: Department of Economics and Social Affairs, https://www.un.org/development/desa/pd/sites/www.un.org.development.desa.pd/files/undesa_pd_2022_household_size_composition.pdf (2022).
104. United Nations. *Household Size and Composition Around the World*. Washington D.C.: Department of Economics and Social Affairs, https://www.un.org/en/development/desa/population/publications/pdf/ageing/household_size_and_composition_around_the_world_2017_data_booklet.pdf (2017).
105. United Nations Development Program. Mission, goals, and mandate. *Development Program*, https://www.undp.org/about-us (2022).
106. United Nations Development Program. Dimensions and indicators. *Human Development Index*, https://hdr.undp.org/data-center/human-development-index#/indicies/HDI (2022).
107. United Nations Development Program. Documentation and downloads. *Human Development Reports*, https://hdr.undp.org/data-center/documentation-and-downloads (2022).
108. United Nations Food and Agriculture Organizations. *Collecting Livestock Data: A Snapshot of Survey Methods*. Roma: Statistics Division, https://www.fao.org/3/as301e/as301e.pdf (2012).
109. United Nations Food and Agriculture Organizations. Crop and livestock production and utilization. *Data Collection*, https://www.fao.org/statistics/data-collection/en/ (2022).
110. United Nations Food and Agriculture Organizations. Statistical work. *Statistics*, https://www.fao.org/statistics/en/ (2022).
111. World Meteorological Organization. About the agency. *Specialized Agency of the United Nations*, https://public.wmo.int/en/about-us/who-we-are (2022).
112. United Nations. Relative humidity. *Records View*, http://data.un.org/data.aspx?d=clino&f=elementcode%3a11 (2022).
